# Evaluation of urinary tissue inhibitor of metalloproteinase-2 in acute kidney injury: a prospective observational study

**DOI:** 10.1186/s13054-014-0716-5

**Published:** 2014-12-19

**Authors:** Tetsushi Yamashita, Kent Doi, Yoshifumi Hamasaki, Takehiro Matsubara, Takeshi Ishii, Naoki Yahagi, Masaomi Nangaku, Eisei Noiri

**Affiliations:** Department of Nephrology and Endocrinology, The University of Tokyo, 7-3-1 Hongo, Bunkyo-ku, Tokyo, 113-8655 Japan; Department of Emergency and Critical Care Medicine, The University of Tokyo, 7-3-1 Hongo, Bunkyo-ku, Tokyo, 113-8655 Japan; 22nd Century Medical and Research Center, The University of Tokyo, 7-3-1 Hongo, Bunkyo-ku, Tokyo, 113-8655 Japan; Japan Science and Technology Agency/Japan International Cooperation Agency (JST/JICA), Science and Technology Research Partnership for Sustainable Development (SATREPS), 7 Gobancho, Chiyoda-ku, Tokyo, 102-0076 Japan

## Abstract

**Introduction:**

Tissue inhibitor of metalloproteinase-2 (TIMP-2) is an emerging acute kidney injury (AKI) biomarker. We evaluated the performance of urinary TIMP-2 in an adult mixed ICU by comparison with other biomarkers that reflect several different pathways of AKI.

**Methods:**

In this study, we prospectively enrolled 98 adult critically ill patients who had been admitted to the adult mixed ICU. Urinary TIMP-2 and *N*-acetyl-β-d-glucosaminidase (NAG) and plasma neutrophil gelatinase-associated lipocalin (NGAL), interleukin-6 (IL-6) and erythropoietin (EPO) were measured on ICU admission. We evaluated these biomarkers’ capability of detecting AKI and its severity as determined by using the Kidney Disease Improving Global Outcomes serum creatinine criteria, as well as its capacity to predict in-hospital mortality. The impact of sepsis, the leading cause of AKI in ICUs, was also evaluated.

**Results:**

We found AKI in 42 patients (42.9%). All biomarkers were significantly higher in AKI than in non-AKI. In total, 27 patients (27.6%) developed severe AKI. Urinary TIMP-2 was able to distinguish severe AKI from non-severe AKI with an area under the receiver operating characteristic curve (AUC-ROC) of 0.80 (95% confidence interval, 0.66 to 0.90). A total of 41 cases (41.8%) were complicated with sepsis. Although plasma NGAL and IL-6 were increased by sepsis, urinary TIMP-2 and NAG were increased not by sepsis, but by the presence of severe AKI. Plasma EPO was increased only by septic AKI. In-hospital mortality was 15.3% in this cohort. Urinary TIMP-2 and NAG, and plasma NGAL, were significantly higher in non-survivors than in survivors, although plasma IL-6 and EPO were not. Among the biomarkers, only urinary TIMP-2 was able to predict in-hospital mortality significantly better than serum creatinine.

**Conclusion:**

Urinary TIMP-2 can detect severe AKI with performance equivalent to plasma NGAL and urinary NAG, with an AUC-ROC value higher than 0.80. Furthermore, urinary TIMP-2 was associated with mortality. Sepsis appeared to have only a limited impact on urinary TIMP-2, in contrast to plasma NGAL.

**Electronic supplementary material:**

The online version of this article (doi:10.1186/s13054-014-0716-5) contains supplementary material, which is available to authorized users.

## Introduction

Acute kidney injury (AKI), a common problem in intensive care units (ICUs) [1], is associated with significantly increased mortality, hospital length of stay and medical costs [2]. Effective treatment for established AKI other than supportive therapy including dialysis remains unknown [3]. Therefore, it is crucially important to identify patients who are expected to develop AKI and prevent AKI if possible. For this purpose, many AKI biomarkers, including neutrophil gelatinase-associated lipocalin (NGAL), interleukin-18 (IL-18) and L-type fatty acid-binding protein (L-FABP), have been investigated [4-6]. Recently, tissue inhibitor of metalloproteinase-2 (TIMP-2) was reported as an emerging biomarker for predicting severe AKI in critically ill patients [7,8]. In cells of various different types, including cells in renal tubules and glomeruli, TIMP-2 is expressed constitutively [9]. Reportedly, TIMP-2 is involved with G_1_ cell cycle arrest during the early phases of cell injury [10]. Renal tubular cells enter a short period of G_1_ cell cycle arrest following renal ischemic insult [11]. Therefore, enhanced TIMP-2 expression can be expected in the pathological condition of AKI.

A clinical evaluation revealed that urinary TIMP-2 was not inferior to any other biomarker, especially in patients with sepsis [7]. The authors of a multinational prospective observational report described that the most frequent contributing factor to AKI is sepsis, which is observed at a rate of approximately 50% [12]. Authors of other reports have described that 45% to 70% of all AKIs are associated with sepsis [13-15]. It is also widely recognized that patients with both sepsis and AKI have an unacceptably high mortality rate [13]. Reportedly, inflammatory cytokine interleukin-6 (IL-6) was increased in septic AKI patients [16-18], and plasma NGAL detected septic AKI along with endotoxin activity assay [19]. Hypoxic insult is assumed to play a crucially important role in AKI, based on findings obtained from basic research [20]. Erythropoietin (EPO) was shown to have non-hematopoietic tissue-protective effects in animal AKI models [21-23]. Although a previous clinical trial revealed no protective effect of EPO against AKI [24], little is known about whether the blood EPO level is useful to detect renal hypoxic injury or to monitor AKI severity.

This study was conducted to evaluate the performance of urinary TIMP-2 in an adult mixed ICU by comparison with other biomarkers used to monitor different pathways: plasma NGAL and IL-6 for inflammation, plasma EPO for hypoxia and urinary *N*-acetyl-β-d-glucosaminidase (NAG) for renal tubular epithelial injury. These comparisons of different biomarkers were expected to reveal the contributing pathophysiological pathway to AKI development and mortality. We also evaluated the influence of sepsis and the prediction of mortality in each biomarker. Although researchers in a multicenter international study previously evaluated the performance of urinary TIMP-2 with a larger population, the present study includes the important strength of comparing urinary TIMP-2 with other biomarkers that are not limited to AKI and can be used to monitor different mechanisms of diseases. Moreover, this additional validation study is the first conducted by a research group independent from the group that originally reported the performance of urinary TIMP-2 [7,8].

## Materials and methods

### Participants and study design

All patients in this study were older than 20 years of age. All had been admitted to ICUs other than the coronary care unit of The University of Tokyo Hospital. In this study, we enrolled 100 consecutive ICU patients from July 2011 to October 2011. Patients were excluded if they had end-stage renal disease or if any of their data were missing. One patient with end-stage renal disease and another patient who had insufficient data were excluded from this cohort. The study protocol was approved by The University of Tokyo Institutional Review Board. Informed consent was obtained from each participant or the participant’s family.

The following clinical variables were evaluated: age, sex, complication of diabetes mellitus and/or hypertension, surgical state, serum creatinine and blood lactate at ICU admission, Acute Physiology and Chronic Health Evaluation (APACHE) II score [25] and non-renal APACHE II score (APACHE II score without renal score), ICU length of stay and in-hospital mortality. This information was obtained from medical records. AKI was determined by changes in serum creatinine according to the Kidney Disease Improving Global Outcomes (KDIGO) criteria for AKI [3] from ICU admission to 7 days later. AKI was defined as an increase in serum creatinine by 0.3 mg/dl within 48 hours or an increase in serum creatinine to 1.5 times baseline. Baseline serum creatinine was defined as the minimum among the outpatient values measured within 6 months before hospital admission, the inpatient value before ICU admission and the last value before hospital discharge. For a patient with no creatinine measurement within 6 months before ICU admission, the baseline was defined as the minimum among the last value before hospital discharge and the estimated value using the Modification of Diet in Renal Disease equation for Japan [26] for the lower end of the reference range (that is, 75 ml/min/1.73 m^2^) as the KDIGO guidelines suggest. Severe AKI was defined as KDIGO stages 2 and 3. Late-onset AKI was defined as follows: no AKI diagnosis was made at ICU admission, but serum creatinine increased to meet the criteria or renal replacement therapy was started within 1 week. Progression of AKI was defined as worsening of the AKI stage (from non-AKI to AKI of any stage, from stage 1 to either stage 2 or stage 3, or from stage 2 to stage 3). The diagnosis of sepsis was made according to the American College of Chest Physicians and the Society of Critical Care Medicine Consensus Conference Committee guidelines [27].

### Biomarker measurement

Paired urine and blood samples were collected at the time of ICU admission. Plasma and urine supernatants were frozen after centrifugation and were stored at −80°C until measurements were taken. Urinary TIMP-2 and NAG and plasma NGAL, IL-6, and EPO were measured. Urinary TIMP-2 and plasma IL-6 were measured using research assays based on enzyme-linked immunosorbent assay (R&D Systems, Minneapolis, MN, USA; Toray Industries, Kamakura, Japan). Urinary NAG was measured at The University of Tokyo Hospital Clinical Laboratory using the 4-HP-NAG substrate method (L-Type NAG; Wako Pure Chemical Industries, Osaka, Japan). Plasma NGAL was determined (Triage NGAL Device; Alere Medical, San Diego, CA, USA) as described previously [28]. Plasma EPO was measured using a human hypoxia multiplex kit (Meso Scale Discovery, Rockville, MD, USA) and a Sector Imager (MSD 2400; Meso Scale Discovery) according to the manufacturer’s instructions.

### Statistical analyses

For this study, data were expressed as mean ± standard deviation and as median (interquartile range) when the data were not normally distributed. Continuous variables were compared using the Wilcoxon rank-sum test or Kruskal–Wallis test for one-way analysis of variance. When the Kruskal–Wallis test for one-way analysis of variance showed statistical significance, a *post hoc* Steel–Dwass test was subsequently conducted. Categorical variables were described as proportions and were compared using either the Pearson χ^2^ test or the two-sided Fisher’s exact test. The biomarker performance was assessed using receiver operating characteristic (ROC) curve analysis. Comparisons of ROC curves were performed as reported previously [29,30]. To evaluate the impact of the biomarkers evaluated in this study of severe AKI detection and in-hospital mortality prediction, we determined the continuous net reclassification improvement (NRI) index and the integrated discrimination improvement (IDI) index [31-33]. Calculations were conducted using statistical analysis software (JMP Pro 11.0.0; SAS Institute, Cary, NC, USA) and R 3.1.1 (R Foundation for Statistical Computing, Vienna, Austria). The null hypothesis was rejected for *P* < 0.05.

## Results

### Patient characteristics and outcomes

Table 1 presents baseline clinical data and outcomes of the enrolled patients. AKI occurred in 42 (42.9%) cases including 27 severe AKI (KDIGO stages 2 and 3). Compared with the non-AKI patients, the patients with AKI were older and more frequently had diabetes complications. Forty-one cases (41.8%) were complicated with sepsis. Sepsis was associated significantly with AKI. The APACHE II scores in the AKI group were significantly higher than in the non-AKI group. In-hospital mortality was 15.3% in the overall cohort. The AKI group showed significantly higher in-hospital mortality.

**Table 1:** **Baseline clinical data and outcomes of enrolled patients** ^**a**^

	**Non-AKI (** ***N*** **= 56)**	**AKI (** ***N*** **= 42)**	***P*** **-value**
Age (yr)	63.0 (43.0 to 75.8)	69.0 (60.0 to 75.3)	0.05
Males, *n* (%)	37 (66.1)	34 (81.0)	0.12
Diabetes, *n* (%)	6 (10.7)	15 (35.7)	0.01
Hypertension, *n* (%)	20 (35.7)	20 (47.6)	0.30
Elective surgical, *n* (%)	12 (21.4)	2 (4.8)	0.02
Emergency surgical, *n* (%)	9 (16.1)	4 (9.5)	0.39
Medical, *n* (%)	35 (62.5)	36 (85.7)	0.01
Sepsis, *n* (%)	15 (26.8)	26 (61.9)	0.001
Baseline serum creatinine (mg/dl)	0.65 (0.46 to 0.79)	0.68 (0.49 to 0.91)	0.23
Measured in outpatient department, *n* (%)	15 (28.6)	13 (31.0)	0.42
Measured on general ward before ICU admission, *n* (%)	7 (12.5)	4 (9.5)	
Measured just before hospital discharge, *n* (%)	30 (53.6)	18 (42.9)	
Estimated by MDRD formula, *n* (%)	4 (7.1)	7 (16.7)	
Serum creatinine on ICU admission (mg/dl)	0.70 (0.52 to 0.89)	1.46 (0.99 to 2.87)	<0.0001
APACHE II score	14.5 ± 8.1	27.0 ± 8.5	<0.0001
ICU length of stay (days)	5 (3 to 8)	9 (5 to 17)	0.001
In-hospital mortality, n (%)	4 (7.1)	11 (26.2)	0.01

^a^AKI, Acute kidney injury; APACHE, Acute Physiology and Chronic Health Evaluation; ICU, Intensive care unit. Baseline serum creatinine was defined as the minimum among the outpatient values measured within 6 months before hospital admission, the inpatient value before ICU admission and the last value before hospital discharge. For a patient with no creatinine measurement within 6 months before ICU admission, the baseline was defined as the lesser of the last value before hospital discharge and the estimated value using the Modification of Diet in Renal Disease (MDRD) equation.

Among 42 patients with AKI, 9 patients (21%) were not diagnosed as having AKI at ICU admission but showed sufficient serum creatinine elevation for AKI diagnosis within 1 week thereafter (late-onset AKI). Of the 42 patients with AKI, 16 (38%) showed further increase of AKI severity after ICU admission (progression of AKI).

### Acute kidney injury detection by biomarkers

**Figure 1:**
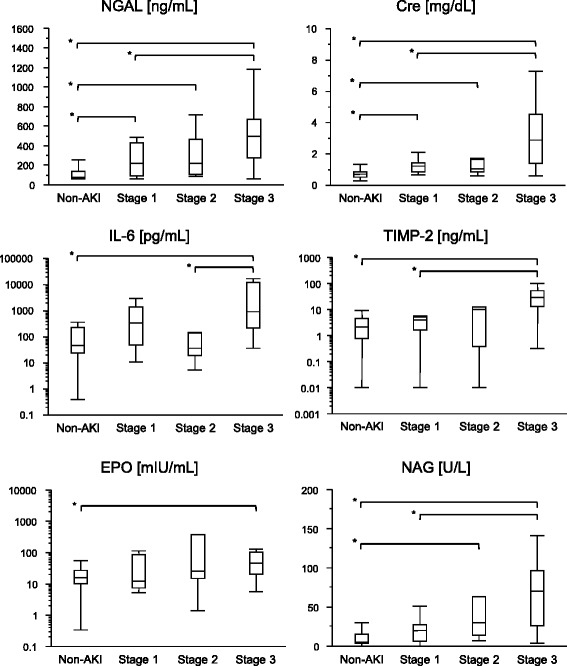
**Biomarker values grouped by acute kidney injury severity.** Values of the evaluated biomarkers measured at intensive care unit admission are shown in each acute kidney injury (AKI) severity category (non-AKI (*n* = 56), stage 1 (*n* = 15), stage 2 (*n* = 7), stage 3 (*n* = 20)). *P < 0.05. Cre, Creatinine; EPO, Erythropoietin; IL-6, Interleukin-6; NAG, *N*-acetyl-β-d-glucosaminidase; NGAL, Neutrophil gelatinase-associated lipocalin; TIMP-2, Tissue inhibitor of matrix metalloproteinase-2.

All biomarkers were significantly higher in the AKI group than in the non-AKI group (Table 2). Plasma NGAL and urinary NAG appeared to be increased along with the severity of AKI (Figure 1). ROC analysis for detecting AKI revealed that plasma NGAL and urinary NAG showed higher area under the ROC curve (AUC-ROC) values than the other biomarkers did (Table 3). Similar results were observed when ROC analysis was conducted for detection of severe AKI (KDIGO stages 2 and 3). For detecting late-onset AKI, only plasma NGAL showed AUC-ROC values higher than 0.70 with statistical significance. All the evaluated biomarkers except plasma EPO were able to detect AKI progression (Additional file 1: Table S1 and Additional file 2: Table S2).

**Table 2:** **Biomarkers in acute kidney injury** ^**a**^

	**Non-AKI (** ***N*** **= 56)**	**AKI (** ***N*** **= 42)**	***P*** **-value**
Plasma NGAL (ng/ml)	80 (60 to 142)	322 (157 to 540)	<0.0001
Plasma IL-6 (pg/ml)	45.1 (22.9 to 226.3)	322.4 (70.3 to 5150.6)	0.0002
Plasma EPO (mIU/ml)	16.1 (9.9 to 28.5)	27.8 (10.2 to 106.0)	0.02
Urinary TIMP-2 (ng/ml)	2.08 (0.72 to 4.59)	10.85 (2.23 to 34.60)	<0.0001
Urinary NAG (U/L)	5.9 (3.1 to 15.0)	31.8 (14.1 to 71.4)	<0.0001

^a^AKI, Acute kidney injury; EPO, Erythropoietin; IL, Interleukin; NAG, *N*-acetyl-β-d-glucosaminidase; NGAL, Neutrophil gelatinase-associated lipocalin; TIMP-2, Tissue inhibitor of matrix metalloproteinase-2.

**Table 3:** **Area under the receiver operating characteristic curve values for acute kidney injury detection by biomarkers** ^**a**^

	**AKI**	**Severe AKI**	**Septic AKI**	**Septic severe AKI**
Plasma NGAL	0.84 (0.74 to 0.91)^b^	0.87 (0.76 to 0.93)^b^	0.94 (0.88 to 0.97)^c^	0.92 (0.84 to 0.96)^d^
Plasma IL-6	0.72 (0.61 to 0.81)	0.70 (0.57 to 0.80)	0.88 (0.79 to 0.93)^d^	0.84 (0.74 to 0.91)
Plasma EPO	0.63 (0.51 to 0.74)	0.71 (0.57 to 0.82)	0.65 (0.52 to 0.77)	0.78 (0.66 to 0.87)
Urinary TIMP-2	0.75 (0.63 to 0.84)	0.81 (0.66 to 0.90)	0.78 (0.65 to 0.88)	0.84 (0.68 to 0.92)
Urinary NAG	0.84 (0.73 to 0.90)^e^	0.88 (0.78 to 0.94)^b^	0.84 (0.72 to 0.91)^d^	0.90 (0.81 to 0.95)^d^

^a^AKI, Acute kidney injury; EPO, Erythropoietin; IL, Interleukin; NAG, *N*-acetyl-β-d-glucosaminidase; NGAL, Neutrophil gelatinase-associated lipocalin; TIMP-2, Tissue inhibitor of matrix metalloproteinase-2. ^b^ *P* < 0.05 vs. IL-6 and EPO; ^c^ *P* < 0.05 vs. EPO, TIMP-2 and NAG; ^d^ *P* < 0.05 vs. EPO; ^e^ *P* < 0.05 vs. EPO and TIMP-2. Data are areas under the receiver operating characteristic curve with 95% confidence intervals.

### Biomarkers in septic acute kidney injury

**Figure 2:**
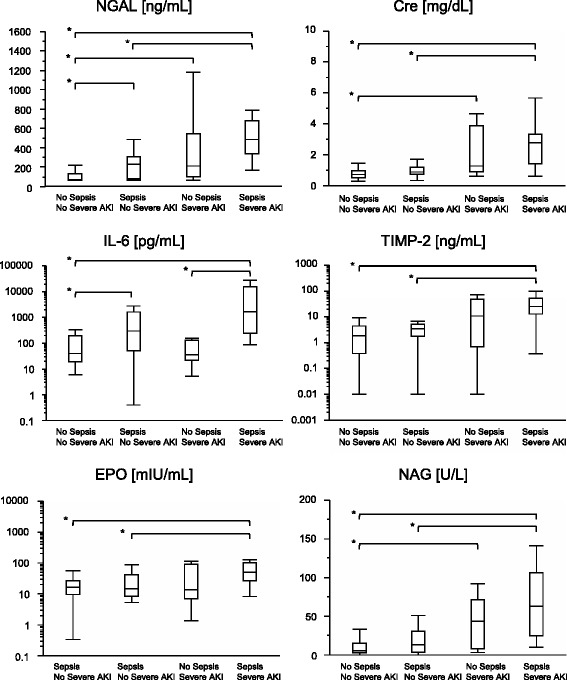
**Biomarker values in septic severe acute kidney injury.** Values of the evaluated biomarkers in the four groups categorized by sepsis and severe acute kidney injury (AKI) complication are shown. Non-septic, non-severe AKI (*n* = 49), septic non-severe AKI (*n* = 22), non-septic severe AKI (*n* = 8) and septic severe AKI (*n* = 19). *P < 0.05. Cre, Creatinine; EPO, Erythropoietin; IL-6, Interleukin-6; NAG, *N*-acetyl-β-d-glucosaminidase; NGAL, Neutrophil gelatinase-associated lipocalin; TIMP-2, Tissue inhibitor of matrix metalloproteinase-2.

Sepsis and severe AKI synergistically worsen the outcomes of critically ill patients in ICUs. Therefore, we further evaluated the performance of biomarkers for detecting severe septic AKI. In accordance with previous reports, plasma NGAL and IL-6 were increased by sepsis, irrespective of AKI complication. However, urinary TIMP-2 and NAG were not influenced by sepsis. Plasma EPO was increased only in AKI cases that were complicated with sepsis (Figure 2). It is noteworthy that plasma NGAL showed a remarkably high AUC-ROC value of 0.94 (95% confidence interval, 0.88 to 0.97) and 0.92 (0.84 to 0.96) for detecting septic AKI and septic severe AKI, respectively (Table 3). Subanalysis of the septic and non-septic populations revealed that the performance of biomarkers evaluated by ROC analysis in the septic population was better than that in the non-septic population (Additional file 3: Table S3). We also determined the NRI and IDI indices in septic and non-septic AKI (Additional file 4: Table S4). Continuous NRI and IDI revealed that the biomarkers that are less influenced by sepsis—TIMP-2, NAG and EPO—improved prediction of severe AKI in the septic population when added to the clinical model, which incorporated age, sex, complication of diabetes, medical admission and serum creatinine.

### Mortality prediction by biomarkers measured at ICU admission

Urinary TIMP-2 and NAG and plasma NGAL were significantly higher in non-survivors than in survivors, although plasma IL-6 and EPO were not associated significantly with mortality (Table 4). Urinary TIMP-2 showed the highest AUC-ROC values for 7-day and in-hospital mortality among the measured biomarkers, including serum creatinine (Table 5). The AUC-ROC values for 7-day mortality of urinary TIMP-2 and NAG were significantly superior to those of creatinine, whereas only urinary TIMP-2 showed a significantly higher AUC-ROC value for in-hospital mortality than that of creatinine.

**Table 4:** **Biomarkers and in-hospital mortality**

	**Survivors (** ***N*** **= 83)**	**Non-survivors (** ***N*** **= 15)**	***P*** **-value**
Plasma NGAL (ng/ml)	111 (60 to 282)	269 (91 to 583)	0.03
Plasma IL-6 (pg/ml)	78.2 (25.1 to 545.5)	342.7 (48.3 to 924.0)	0.06
Plasma EPO (mIU/ml)	17.1 (9.8 to 36.9)	33.6 (10.4 to 88.7)	0.21
Serum creatinine (mg/dl)	0.83 (0.61 to 1.41)	1.04 (0.75 to 2.42)	0.19
Urinary TIMP-2 (ng/ml)	2.8 (0.9 to 6.9)	11.4 (3.8 to 65.7)	0.004
Urinary NAG (U/L)	11.9 (3.5 to 24.9)	33.0 (13.4 to 51.1)	0.01

EPO, Erythropoietin; IL, Interleukin; NAG, *N*-acetyl-β-d-glucosaminidase; NGAL, Neutrophil gelatinase-associated lipocalin; TIMP-2, Tissue inhibitor of matrix metalloproteinase-2.

**Table 5:** **Area under the receiver operating characteristic curve values for mortality prediction by biomarkers**

	**7-day mortality**	**In-hospital mortality**
Plasma NGAL	0.79 (0.67 to 0.88)	0.68 (0.53 to 0.80)
Plasma IL-6	0.82 (0.57 to 0.94)	0.66 (0.50 to 0.79)
Plasma EPO	0.63 (0.38 to 0.90)	0.60 (0.44 to 0.75)
Serum creatinine	0.67 (0.53 to 0.78)	0.61 (0.45 to 0.74)
Urinary TIMP-2	0.83 (0.59 to 0.94)*	0.74 (0.60 to 0.85)*
Urinary NAG	0.80 (0.58 to 0.92)*	0.70 (0.54 to 0.82)

EPO, Erythropoietin; IL, Interleukin; NAG, *N*-acetyl-β-d-glucosaminidase; NGAL, Neutrophil gelatinase-associated lipocalin; TIMP-2, Tissue inhibitor of matrix metalloproteinase-2. *p < .05 vs. serum creatinine.

### Improvement of AKI detection and mortality prediction by addition of biomarkers to the clinical model

In the clinical model, we incorporated age, sex, complications of diabetes and sepsis, medical admission, and serum creatinine. Addition of the five biomarkers evaluated in this study to the clinical model did not increase AUC-ROC values significantly. We also determined the continuous NRI and the IDI indices. Addition of urinary TIMP-2 or NAG significantly improved risk prediction of severe AKI when evaluated using continuous NRI and IDI. Addition of urinary TIMP-2 also showed significant improvement of in-hospital mortality as evaluated by continuous NRI (Table 6).

**Table 6:** **AUC-ROC, continuous net reclassification improvement index and integrated discrimination improvement index when each biomarker was added to the clinical model** ^**a**^

	**Severe AKI**			**In-hospital mortality**		
	**AUC-ROC**	**Continuous NRI**	**IDI**	**AUC-ROC**	**Continuous NRI**	**IDI**
Clinical model	0.87 (0.76 to 0.94)			0.72 (0.57 to 0.83)		
+ NGAL	0.89 (0.77 to 0.95)	25 (−19 to 69)	0.03 (−0.00 to 0.07)	0.72 (0.58 to 0.83)	24 (−31 to 79)	0.00 (−0.01 to 0.01)
+ IL-6	0.88 (0.76 to 0.94)	−8 (−51 to 35)	0.00 (−0.00 to 0.01)	0.72 (0.58 to 0.83)	10 (−44 to 64)	0.01 (−0.02 to 0.04)
+ EPO	0.88 (0.75 to 0.94)	34 (−10 to 78)	0.01 (−0.01 to 0.03)	0.72 (0.57 to 0.83)	−13 (−68 to 42)	0.00 (−0.00 to 0.01)
+ TIMP-2	0.89 (0.76 to 0.95)	41 (1 to 82)^b^	0.04 (0.00 to 0.08)^b^	0.76 (0.64 to 0.86)	64 (17 to 109)^b^	0.03 (−0.01 to 0.06)
+ NAG	0.93 (0.82 to 0.97)	79 (38 to 119)^b^	0.13 (0.05 to 0.21)^b^	0.74 (0.61 to 0.84)	24 (−30 to 78)	0.01 (−0.01 to 0.03)

^a^AKI, Acute kidney injury; AUC-ROC, Area under the receiver operating characteristic curve; EPO, Erythropoietin; IDI, Integrated discrimination improvement index; IL, Interleukin; NAG, *N*-acetyl-β-d-glucosaminidase; NGAL, Neutrophil gelatinase-associated lipocalin; NRI, Net reclassification improvement index; TIMP-2, Tissue inhibitor of matrix metalloproteinase-2. ^b^ *P* < 0.05 vs. clinical model.

## Discussion

This study demonstrates that urinary TIMP-2 can detect severe AKI with performance as good as that of plasma NGAL and urinary NAG, with an AUC-ROC value higher than 0.80. We observed no significant impact of sepsis on urinary TIMP-2, although the authors of a previous report presented a better prediction of AKI by the combination of TIMP-2 and insulin-like growth factor-binding protein-7 (IGFBP-7) in septic subjects than in post-surgery subjects [7]. The enrolled patients treated in a mixed ICU in the present study might have had not only AKI but also several other organ injuries. In addition to AKI detection, urinary TIMP-2 was able to predict mortality better than serum creatinine. These data, obtained with a heterogeneous ICU population in the present study, validate previous reports that demonstrated the clinical significance of measuring urinary TIMP-2 [7,8,34] and confirmed its generalizability for clinical translation.

Actually, TIMP-2 has been identified as a potential new AKI biomarker by examination of over 300 markers with a heterogeneous AKI cohort comprising sepsis, shock, major surgery and trauma [7]. Together with TIMP-2, IGFBP-7 was also found to be the best-performing marker in the discovery study. These two molecules are reportedly involved with cell cycle arrest at G_1_ phase [10,35,36]. Therefore, the utility of TIMP-2 and IGFBP-7 suggests a crucial role of cell cycle regulation in the pathogenesis of AKI. Recently, in another independent study of urine proteome analysis using gel electrophoresis and mass spectrometry, researchers identified IGFBP-7 as a novel prognostic marker for AKI [37]. Although urinary IGFBP-7 showed performance similar to NGAL in terms of AKI detection and reflection of AKI severity in an independent verification group of 28 patients with AKI and 12 control patients without AKI, urinary NGAL appeared to predict mortality better than IGFBP-7 did. In the present study, we did not measure urinary IGFBP-7. Further validation studies must be undertaken to confirm the utility of the combination of urinary TIMP-2 and IGFBP-7.

Especially for critically ill patients treated in ICUs for non-surgical conditions, sepsis is the most important factor affecting their prognosis. As also reported in earlier studies [19,38-40], NGAL was able to detect septic AKI with high AUC-ROC values, above 0.90, in the present study. The results of the present study show that IL-6 was increased not by AKI alone, but also by septic AKI, although plasma EPO was increased only by septic AKI. Urinary TIMP-2 and NAG were elevated in AKI, irrespective of sepsis complication. These distinct characteristics of the five examined biomarkers will enable discrimination of the etiologies of AKI. The 10th Acute Dialysis Quality Initiative (ADQI) Consensus Conference recommended that the etiology of AKI should be ascertained by measuring several different biomarkers that help differentiate AKI of uncertain etiology [41]. Further studies must be conducted to determine the specificity of damage and biomarkers for individual disease states.

It is noteworthy that only urinary TIMP-2 showed better prediction of in-hospital mortality among the evaluated biomarkers compared with serum creatinine (Table 5). This feature of new AKI biomarkers has recently been addressed. One meta-analysis showed that blood and urinary NGAL can detect patients who have increased risk of adverse outcomes including mortality, even in the absence of sufficient serum creatinine increase for AKI diagnosis [42]. Another report described a better prediction of mortality of ICU patients by urinary NGAL and L-FABP than that by serum creatinine [40]. These observations suggest that new AKI biomarkers, including TIMP-2, can detect renal structural damage independently from functional changes shown by serum creatinine elevation and that a combination of kidney functional and damage markers enable stratification of patients with AKI at risk for poor outcomes [43].

Several limitations might affect the results obtained from this study. First, this study was conducted at a single center. The number of patients analyzed was insufficiently large. Evaluations in multicenter ICUs with larger cohorts should be conducted to verify our findings. Second, most AKI cases (79%) were diagnosed as AKI on ICU admission, which might indicate that we were unable to enroll proper patients with an early phase of AKI, where novel biomarkers might have had more value than creatinine. Third, we evaluated AKI and sepsis, but did not evaluate their mutual cause-and-effect relationship. Although the pathophysiological mechanisms of sepsis-induced AKI have been investigated widely [44], sepsis can be not only a cause but also a consequence of AKI in a clinical setting. In a multicenter observational study of AKI, researchers reported the clinical consequences of sepsis with AKI [45]. Among the 611 patients with AKI, 174 patients (28%) had sepsis before AKI and 243 patients (40%) developed sepsis after AKI. The relationship of cause and effect between AKI and sepsis can affect biomarker behavior.

## Conclusions

A new urine biomarker, TIMP-2, can detect severe AKI with performance as good as that of plasma NGAL and urinary NAG, with an AUC-ROC value higher than 0.80. In addition, urinary TIMP-2 was associated with mortality. Sepsis appeared to have a limited impact on urinary TIMP-2, in contrast to plasma NGAL. These distinct features of biomarkers might enable the evaluation of the contribution of sepsis to AKI development.

## Key messages

- Urinary TIMP-2 was increased, especially in severe AKI, and was associated with mortality.
- Sepsis had no significant impact on urinary TIMP-2 and NAG, although plasma NGAL and IL-6 were increased by sepsis and AKI.
- Distinct characteristics of respective biomarkers might be helpful to differentiate the AKI etiology.

## Additional files

Additional file 1:
**Biomarkers in established AKI, late-onset AKI and progression of AKI.**
Additional file 2:
**AUC-ROC values for detection of established AKI, late-onset AKI or progression of AKI.**
Additional file 3:
**AUC-ROC values in subanalysis of septic and non-septic population when each biomarker is added to the clinical model.**
Additional file 4:
**Continuous NRI and IDI in subanalysis of septic and non-septic population when each biomarker is added to the clinical model.**

## Abbreviations

ADQI: Acute Dialysis Quality Initiative
AKI: Acute kidney injury
APACHE: Acute Physiology and Chronic Health Evaluation
AUC-ROC: Area under the receiver operating characteristic curve
EPO: Erythropoietin
ICU: Intensive care unit
IDI: Integrated discrimination improvement
IGFBP-7: Insulin-like growth factor-binding protein-7
IL: Interleukin
KDIGO: Kidney Disease Improving Global Outcomes
L-FABP: L-type fatty acid-binding protein
NAG: *N*-acetyl-β-d-glucosaminidase
NGAL: Neutrophil gelatinase-associated lipocalin
NRI: Net reclassification improvement
ROC: Receiver operating characteristic
TIMP-2: Tissue inhibitor of metalloproteinase-2
