# Supplementary material for: Evaluation of urinary tissue inhibitor of metalloproteinase-2 in acute kidney injury: a prospective observational study
Source: Crit Care. 2014 Dec 19;18(6):716. doi: 10.1186/s13054-014-0716-5 (PMC4300076; doi:10.1186/s13054-014-0716-5)
Supplement: Additional file 1: — Biomarkers in established AKI, late-onset AKI and progression of AKI. [file 13054_2014_716_MOESM1_ESM.pdf]

Supplementary Table 1. Biomarkers in established AKI, late-onset AKI, and progression of AKI

	Non-AKI (N=56)	Established AKI (N=33)	Late-onset AKI (N=9)	Progression of AKI (N=16)
Plasma NGAL (ng/mL)	80 (60 to 142)	400 (227 to 622)[a]	196 (101 to 390)[a]	261 (124 to 469)[a]
Plasma IL-6 (pg/mL)	45.1 (22.9 to 226.3)	500.6 (62.5 to 5209.6)[a]	294.4 (68.9 to 10348.2)[a]	318.5 (120.1 to 14371.8)[a]
Plasma EPO (mIU/mL)	16.1 (9.9 to 28.5)	28.7 (11.4 to 109.4)[a]	26.9 (5.85 to 97.9)	33.1 (7.9 to 83.1)
Urinary TIMP-2 (ng/mL)	2.08 (0.72 to 4.59)	12.64 (2.45 to 35.83)[a]	3.94 (1.75 to 28.75)	8.92 (2.37 to 47.41)[a]
Urinary NAG (U/L)	5.9 (3.1 to 15.0)	36.1 (15.7 to 76.1)[a]	26.4 (6.0 to 44.4)[a]	33.9 (9.7 to 97.7)[a]

[a]$p < .05$ vs. Non-AKI
