# Supplementary material for: Evaluation of urinary tissue inhibitor of metalloproteinase-2 in acute kidney injury: a prospective observational study
Source: Crit Care. 2014 Dec 19;18(6):716. doi: 10.1186/s13054-014-0716-5 (PMC4300076; doi:10.1186/s13054-014-0716-5)
Supplement: Additional file 2: — AUC-ROC values for detection of established AKI, late-onset AKI or progression of AKI. [file 13054_2014_716_MOESM2_ESM.pdf]

Supplementary Table 2. AUC-ROC values for detection of established AKI, late-onset AKI, or progression of AKI

	Established AKI (*N*=33)	Late-onset AKI (*N*=9)	Progression of AKI (*N*=16)
Plasma NGAL	0.84 (0.72 to 0.91)[a]	0.77 (0.55 to 0.90)	0.76 (0.60 to 0.87)
Plasma IL-6	0.70 (0.57 to 0.80)	0.72 (0.49 to 0.87)	0.74 (0.59 to 0.85)
Plasma EPO	0.65 (0.53 to 0.76)	0.54 (0.27 to 0.79)	0.58 (0.39 to 0.75)
Urinary TIMP-2	0.75 (0.62 to 0.85)	0.66 (0.44 to 0.83)	0.73 (0.55 to 0.85)
Urinary NAG	0.83 (0.72 to 0.90)[a]	0.75 (0.48 to 0.91)	0.77 (0.58 to 0.89)[b]

[a]$p < .05$ vs. IL-6 and EPO, [b]$p < .05$ vs. EPO
