# Supplementary material for: Evaluation of urinary tissue inhibitor of metalloproteinase-2 in acute kidney injury: a prospective observational study
Source: Crit Care. 2014 Dec 19;18(6):716. doi: 10.1186/s13054-014-0716-5 (PMC4300076; doi:10.1186/s13054-014-0716-5)
Supplement: Additional file 3: — AUC-ROC values in subanalysis of septic and non-septic population when each biomarker is added to the clinical model. [file 13054_2014_716_MOESM3_ESM.pdf]

Supplementary Table 3. Subanalysis of septic and non-septic population

	Severe AKI (*N*=19) in septic patients (*N*=41)	Severe AKI (*N*=8) in non-septic patients (*N*=57)
Clinical Model	0.91 (0.76 to 0.97)	0.85 (0.65 to 0.95)
+ NGAL	0.91 (0.77 to 0.97)	0.83 (0.60 to 0.94)
+ IL-6	0.91 (0.75 to 0.97)	0.86 (0.69 to 0.95)
+ EPO	0.96 (0.86 to 0.99)	0.86 (0.66 to 0.95)
+ TIMP-2	0.96 (0.86 to 0.99)	0.86 (0.70 to 0.95)
+ NAG	0.99 (0.92 to 1.00)	0.88 (0.68 to 0.96)

AUC-ROC values for severe AKI detection when each biomarker is added to the clinical model.
Clinical model incorporates age, sex, complication of diabetes, medical admission, and serum creatinine.
No significant difference is found.
