# Supplementary material for: Evaluation of urinary tissue inhibitor of metalloproteinase-2 in acute kidney injury: a prospective observational study
Source: Crit Care. 2014 Dec 19;18(6):716. doi: 10.1186/s13054-014-0716-5 (PMC4300076; doi:10.1186/s13054-014-0716-5)
Supplement: Additional file 4: — Continuous NRI and IDI in subanalysis of septic and non-septic population when each biomarker is added to the clinical model. [file 13054_2014_716_MOESM4_ESM.pdf]

Supplementary Table 4. Subanalysis of septic and non-septic population

	Severe AKI (*N*=19) in septic patients (*N*=41)		Severe AKI (*N*=8) in non-septic patients (*N*=57)	
	Continuous NRI	IDI	Continuous NRI	IDI
Clinical Model				
+ NGAL	-9 (-68 to 51)	0.00 (-0.02 to 0.03)	56 (-17 to 128)	0.07 (-0.00 to 0.15)
+ IL-6	23 (-37 to 84)	0.01 (-0.02 to 0.03)	93 (40 to 147)[a]	0.05 (-0.07 to 0.16)
+ EPO	75 (18 to 131)[a]	0.12 (0.02 to 0.21)[a]	10 (-64 to 85)	0.00 (-0.03 to 0.04)
+ TIMP-2	103 (51 to 156)[a]	0.11 (0.00 to 0.21)[a]	-10 (-85 to 64)	0.00 (-0.04 to 0.05)
+ NAG	179 (151 to 207)[a]	0.30 (0.15 to 0.46)[a]	14 (-60 to 89)	0.06 (-0.04 to 0.16)

For continuous NRI and IDI, each model is made by adding each biomarker to the clinical model, which incorporated age, sex, complication of diabetes, medical admission, and serum creatinine.

[a]$p < .05$ vs. clinical model
